# Independent and joint associations of sleep duration and physical activity with cardiovascular disease: a cross-sectional study of 1.6 million US adults

**DOI:** 10.3389/fpubh.2026.1886068

**Published:** 2026-07-28

**Authors:** Yuqin Jin, Zheng Gong, Lei Ming, Jianhua Gu, Xi Zhao

**Affiliations:** 1 Department of Radiology, Shandong First Medical University Affiliated Tumor Hospital, Jinan, China; 2 Department of Emergency Medicine, Qilu Hospital of Shandong University, Jinan, China; 3 Department of Radiology, The First Affiliated Hospital of Shandong First Medical University & Shandong Provincial Qianfoshan Hospital, Shandong Medicine and Health Key Laboratory of Abdominal Medical Imaging, Jinan, China; 4 Key Laboratory of Endocrine Glucose & Lipids Metabolism and Brain Aging, Ministry of Education, Department of Endocrinology and Metabolism, Shandong Provincial Hospital Affiliated to Shandong First Medical University, Jinan, China

**Keywords:** behavioral risk factor surveillance system, cardiovascular disease, inverse probability weighting, physical activity, sleep duration

## Abstract

**Background:**

Sleep duration and physical activity are intertwined determinants of cardiovascular health, yet their joint impact remains unclear. This study evaluated their independent, joint, and interactive associations with cardiovascular disease using a rigorous confounding adjustment framework.

**Methods:**

We analyzed pooled data from 1,577,591 US adults in the Behavioral Risk Factor Surveillance System (2014–2022). Inverse probability of treatment weighting and doubly robust logistic regression were used. Additive interaction was assessed via RERI.

**Results:**

Both short sleep (odds ratio, 1.26; 95% confidence interval, 1.22–1.30) and long sleep (odds ratio, 1.17; 95% confidence interval, 1.13–1.21) were independently associated with prevalent cardiovascular disease, as was physical inactivity (odds ratio, 1.11; 95% confidence interval, 1.09–1.14). The joint effect of short sleep and inactivity conferred the highest risk (odds ratio, 1.38; 95% confidence interval, 1.31–1.44). The relative excess risk due to interaction was −0.03 (95% confidence interval, −0.11 to 0.06), indicating additive, independent risks without synergy.

**Conclusion:**

By applying a rigorous adjustment framework, this study provides actionable, population-level evidence that moves beyond simple risk factor identification. Public health strategies should integrate sleep hygiene and physical activity into holistic “24-h movement” prescriptions.

## Introduction

Cardiovascular diseases (CVDs) remain the leading cause of disability and mortality worldwide, presenting a sustained challenge to global public health practice. Recent data from the Global Burden of Disease Study 2023 estimate that the number of CVD-related deaths reached 19.2 million globally in 2023, while the number of prevalent cases has more than doubled since 1990 to approximately 626 million (1, 2). Importantly, nearly 80% of this disease burden is estimated to be attributable to modifiable risk factors, suggesting that lifestyle modification remains a primary strategy for prevention (1). Among these factors, sleep duration and physical activity represent two fundamental components of daily behavior that significantly influence cardiovascular health. Growing evidence indicates that these behaviors may modulate cardiovascular risk through shared physiological pathways (3). For example, recent mechanistic studies suggest that sleep disturbances can influence systemic inflammation and neuroimmune signaling (4, 5), while regular physical activity is associated with improved vascular function and reduced chronic inflammation (6).

Despite the established biological plausibility linking sleep and physical activity to cardiovascular health (7, 8), current research paradigms often investigate these behaviors in isolation, overlooking their intrinsic interconnectedness. Health behaviors rarely occur in a vacuum; rather, they cluster within individuals—poor sleep can disrupt circadian rhythms and diminish motivation for exercise, creating a vicious cycle of cardiovascular risk (9–11). While pioneering studies from large-scale cohorts, such as the UK Biobank and the MJ Cohort, have begun to explore the joint impact of these behaviors, existing evidence has predominantly focused on mortality outcomes rather than disease prevalence (12–14). Moreover, prior research has largely relied on categorical analyses, an oversight that has yet to delineate the continuous dose–response relationship between sleep duration and CVD within a physical activity framework (9, 14). Crucially, there remains a paucity of evidence examining the combined burden of this ‘double-burden’ lifestyle on cardiovascular morbidity, which is essential for informing targeted population-level interventions and public health resource allocation. Furthermore, the distribution of these lifestyle behaviors is not uniform across sociodemographic strata; national surveillance data consistently demonstrate that physical inactivity is more prevalent among females and older adults, whereas short sleep duration is disproportionately reported among Black and Hispanic populations. This heterogeneity underscores the necessity of stratified analyses to identify high-risk subgroups for targeted prevention.

To address these knowledge gaps and inform evidence-based public health practice, this study utilized pooled data from five biennial cycles of the Behavioral Risk Factor Surveillance System (BRFSS; 2014, 2016, 2018, 2020, and 2022), a large-scale, nationally representative survey. The primary objectives were: (1) to examine the independent and joint associations of sleep duration and physical activity with CVD prevalence; (2) to characterize the dose–response relationship between continuous sleep duration and cardiovascular risk using restricted cubic splines (RCS); and (3) to examine the consistency of these associations across key sociodemographic and clinical subgroups. By employing a doubly robust IPTW approach and comprehensive subgroup analyses, we aimed to provide not only etiological insights but also quantifiable evidence for public health decision-making. We hypothesized that the co-occurrence of short sleep and physical inactivity would exert a synergistic deleterious effect on cardiovascular health, disproportionately affecting vulnerable sociodemographic subgroups.

## Methods

### Study design and population

This cross-sectional study analyzed pooled data from five biennial cycles (2014, 2016, 2018, 2020, and 2022) of the BRFSS, a nationally representative telephone survey conducted by the Centers for Disease Control and Prevention (CDC) (15). To ensure rigorous confounding control and stable subgroup estimates, we implemented strict exclusion criteria. From the initial cohort, we excluded participants aged <18 years, pregnant individuals, and those with missing data on core sociodemographic weighting variables, cardiovascular outcomes, or primary exposures. A complete-case analysis was performed by further excluding participants with missing covariate data to facilitate propensity score weighting. The numbers of participants excluded at each step are detailed in Supplementary Figure S1; the final analytical sample comprised 1,577,591 respondents. Missingness was primarily driven by household income (*n* = 383,948) and BMI (*n* = 185,709), with smaller contributions from other covariates. As this study utilized de-identified public data, it was exempt from institutional review board review and adhered to STROBE reporting guidelines. To assess the potential impact of the COVID-19 pandemic on our findings, we conducted a sensitivity analysis excluding the 2020 cycle.

### Exposure and outcome assessments

The primary outcome was CVD, defined as a self-reported lifetime diagnosis of myocardial infarction, coronary heart disease, or stroke, based on responses to the BRFSS questionnaire. Self-reported CVD diagnoses have demonstrated good agreement with medical records in previous validation studies (16).

Sleep duration was assessed by the question: “On average, how many hours of sleep do you get in a 24-h period?” and categorized according to established cutoffs from large-scale epidemiological studies: short (<6 h), normal (6–8 h, reference), and long (>8 h) (17).

Physical activity was assessed by the question: “During the past month, did you participate in any leisure-time physical activity?” Responses were dichotomized as Active (yes) versus Inactive (no), consistent with the CDC’s standard analytical framework for BRFSS surveillance data (15).

### Statistical analysis

All analyses accounted for the complex survey design, including stratification, clustering, and sampling weights (15). To mitigate confounding and selection bias for the physical activity exposure, we applied inverse probability of treatment weighting (IPTW) (18). Propensity scores for physical inactivity were estimated using survey-weighted logistic regression conditional on a comprehensive set of sociodemographic, behavioral, and clinical covariates. Sleep duration was not weighted; instead, it was included as a covariate in the propensity score model and as an independent exposure in the outcome model, adjusted through regression. Stabilized weights were trimmed at the 1st and 99th percentiles to minimize variance inflation (19). Covariate balance was assessed using absolute standardized mean differences (SMD), with values <0.10 indicating negligible imbalance (20).

We estimated the associations using three models: Model 1 provided unadjusted estimates; Model 2 utilized conventional multivariable logistic regression adjusted for sociodemographics; and Model 3 employed a doubly robust strategy combining IPTW with regression adjustment for all covariates to maximize confounding control. For physical inactivity, the estimand of interest was the average treatment effect (ATE) in the IPTW-weighted population. For sleep duration, the estimand was a conditional association adjusted for all covariates including the IPTW weights. Additive interaction between short sleep and inactivity was assessed using the Relative Excess Risk due to Interaction (RERI) and the Synergy Index (S) (21). Consistent with epidemiological standards, RERI values significantly deviating from zero indicated additive interaction. We visualized the dose–response relationship between continuous sleep duration and CVD risk stratified by physical activity using restricted cubic splines with three knots (22), based on the IPTW-weighted population.

To assess heterogeneity, we performed prespecified subgroup analyses stratified by key sociodemographic and clinical factors. Multiplicative interactions were tested using the Wald test. Sensitivity analyses included restricting the sample to participants with good-to-excellent self-rated health to minimize reverse causality and calculating the E-value to assess robustness against unmeasured confounding. Variance estimation accounted for both the complex survey design (via survey-weighted robust standard errors) and the trimmed IPTW weights. To evaluate the impact of missing data, we performed a multiple imputation by chained equations (MICE) sensitivity analysis with five imputed datasets and 20 iterations, including all covariates, exposures, and the outcome in the imputation model. Rubin’s rules were used to combine estimates. We repeated Model 2 in each imputed dataset and compared the pooled results with the complete-case estimates. We assessed the calibration of the multivariable-adjusted logistic regression model (Model 2) by grouping participants into deciles of predicted risk and comparing observed versus predicted probabilities of CVD across deciles (23). Well-calibrated predictions are indicated by close agreement between observed proportions and mean predicted probabilities within each decile.

Analyses were conducted in R version 4.3.0 (R Foundation), with 2-sided *p* values <0.05 considered significant.

## Results

### Population characteristics and covariate balance

Among the 1,577,591 participants representing the US adult population, the weighted prevalence of specific risk factors remained stable across the study period (2014–2022), supporting the validity of data pooling (Supplementary Table S1). In the unweighted cohort, physically inactive participants were significantly older and had lower socioeconomic status and a higher burden of comorbidities compared with active participants. For instance, the prevalence of poor general health was nearly three times higher in the inactive group (32.5% vs. 11.6%). Following the application of IPTW, these baseline imbalances were effectively mitigated; the standardized mean differences for all covariates were reduced to below 0.10, indicating that the weighted pseudo-population achieved quasi-randomized balance across covariates (Supplementary Figure S2 and Table 1).

**Table 1:** Baseline characteristics of the study population before and after IPTW adjustment.

Variable	Original cohort (before IPTW)			Balanced cohort (after IPTW)		
	Active group	Inactive group	SMD	Active group	Inactive group	SMD
	(*N* = 1,216,153)	(*N* = 361,438)				
	No. (weighted %)	No. (weighted %)		Weighted %	Weighted %	
Sleep duration			**0.246**			0.05
Normal (6–8 h)	1,025,502 (83.3)	264,798 (73.3)		81.2	79.2	
Short (<6 h)	105,838 (10.2)	55,415 (16.5)		11.6	12.8	
Long (>8 h)	84,813 (6.5)	41,225 (10.2)		7.3	8.1	
Age group			**0.309**			0.107
18–24	68,027 (11.8)	10,178 (6.7)		10.6	8.4	
25–34	141,888 (19.0)	25,783 (13.3)		17.8	16.1	
35–44	168,394 (17.8)	36,948 (15.3)		17.2	16.8	
45–54	201,869 (16.9)	56,220 (18.0)		17.1	17.3	
55–64	256,460 (16.4)	83,142 (20.0)		17.2	18.5	
≥65	379,515 (18.1)	149,167 (26.8)		20.1	22.9	
Sex			**0.116**			0.024
Female	629,595 (47.8)	209,257 (53.6)		49.1	50.3	
Male	586,558 (52.2)	152,181 (46.4)		50.9	49.7	
Race/Ethnicity			**0.192**			0.049
White	960,781 (66.4)	265,150 (59.1)		65.4	65.3	
Black	84,398 (10.6)	34,868 (13.8)		11.4	12	
Hispanic	87,985 (14.5)	36,902 (19.9)		15.2	15.4	
Asian	29,105 (5.2)	6,997 (4.1)		4.7	3.8	
Other	53,884 (3.3)	17,521 (3.1)		3.3	3.5	
Education level			**0.527**			0.081
<High school	52,059 (8.4)	40,709 (19.9)		10.6	12.1	
High school grad	274,935 (24.7)	130,594 (34.5)		26.9	29.2	
Some college	333,664 (32.2)	103,347 (29.3)		31.5	30.7	
College grad	555,495 (34.7)	86,788 (16.4)		30.9	28.1	
Annual household income			**0.518**			**0.11**
<$25,000	226,825 (19.7)	137,125 (38.3)		23.8	27.3	
$25,000–$50,000	285,298 (22.4)	104,352 (27.6)		23.5	25.4	
>$50,000	704,030 (57.9)	119,961 (34.1)		52.7	47.3	
Marital status			**0.126**			0.006
Married	684,149 (53.8)	166,441 (47.5)		52.3	52	
Unmarried/Other	532,004 (46.2)	194,997 (52.5)		47.7	48	
BMI category			**0.296**			0.089
Normal	393,277 (33.2)	82,128 (24.3)		31.2	28.1	
Underweight	16,639 (1.6)	6,693 (2.1)		1.7	1.8	
Overweight	455,380 (36.4)	115,211 (31.5)		35.4	34.6	
Obese	350,857 (28.8)	157,406 (42.1)		31.7	35.5	
Smoking status			**0.247**			0.068
Never	722,685 (61.7)	175,189 (51.4)		59.2	56.2	
Former	343,819 (24.6)	110,229 (26.6)		25.1	26	
Current	149,649 (13.6)	76,020 (22.1)		15.7	17.8	
Lifestyle and health (Yes)						
Binge drinking	190,495 (19.1)	39,384 (13.6)	**0.15**	17.9	16.2	0.047
Health insurance	1,139,923 (90.5)	330,220 (86.3)	**0.131**	89.7	89	0.021
General health (Fair/Poor)	142,735 (11.6)	124,705 (32.5)	**0.521**	16.2	19.7	0.092
Depressive disorder	210,951 (17.1)	95,317 (24.8)	**0.19**	18.9	20.9	0.051
Clinical comorbidities (Yes)						
Diabetes	130,445 (9.0)	78,877 (18.5)	**0.278**	11.1	13.3	0.067
Kidney disease	36,509 (2.4)	23,927 (5.4)	**0.157**	3	3.6	0.03
COPD	71,264 (4.8)	54,706 (12.2)	**0.267**	6.5	7.7	0.046
Asthma	157,340 (13.6)	60,195 (16.2)	0.075	14.2	14.5	0.01
Arthritis	366,265 (22.7)	165,002 (36.4)	**0.305**	25.8	29.2	0.077
Cancer history	195,315 (10.9)	69,627 (14.1)	0.098	11.6	12.5	0.028

BMI, Body Mass Index; COPD, Chronic Obstructive Pulmonary Disease; IPTW, Inverse Probability of Treatment Weighting; No., Number; SMD, Standardized Mean Difference.

Data represent unweighted sample size (No.) and weighted percentage (%) accounting for the complex survey design.

The “Balanced Cohort” percentages are adjusted using trimmed composite weights (survey weight × IPTW weight).

SMD > 0.1 (bolded) indicates potential imbalance between groups. After IPTW adjustment, all covariates achieved satisfactory balance (SMD < 0.1), demonstrating the efficacy of the weighting procedure in creating a pseudo-randomized population.

### Independent and joint associations with cardiovascular disease

In the rigorous doubly robust IPTW analysis (Model 3), both short sleep duration (OR, 1.26; 95% CI, 1.22–1.30) and physical inactivity (OR, 1.11; 95% CI, 1.09–1.14) were independently associated with elevated CVD prevalence. When evaluating their joint impact, the combination of short sleep and physical inactivity conferred the highest risk. Individuals in this “double-hit” category had 38% higher odds of CVD compared with those maintaining a normal sleep duration and active lifestyle (OR, 1.38; 95% CI, 1.31–1.44). This estimate exceeded the risk associated with short sleep alone (OR 1.28) or inactivity alone (OR 1.12), demonstrating a cumulative deleterious effect (Table 2).

**Table 2:** Independent and joint associations of sleep duration and physical activity with cardiovascular disease prevalence.

Exposure	Model 1: Unadjusted	Model 2: Multivariable Adjusted	Model 3: IPTW Analysis
	OR (95% CI)	OR (95% CI)	OR (95% CI)
A. Independent associations^a^			
Sleep duration			
Normal (6–8 h)	1.00 (Reference)	1.00 (Reference)	1.00 (Reference)
Short (<6 h)	1.58 (1.54–1.63)	1.26 (1.21–1.30)	1.26 (1.22–1.30)^b^
Long (>8 h)	1.83 (1.76–1.90)	1.19 (1.14–1.24)	1.17 (1.13–1.21)^b^
Physical activity			
Active	1.00 (Reference)	1.00 (Reference)	1.00 (Reference)
Inactive	2.14 (2.09–2.19)	1.08 (1.05–1.11)	1.11 (1.09–1.14)^c^
B. Joint associations^d^			
Sleep and physical activity			
Normal sleep and active	1.00 (Reference)	1.00 (Reference)	1.00 (Reference)
Normal sleep and inactive	2.14 (2.08–2.20)	1.10 (1.06–1.13)	1.12 (1.09–1.15)
Short sleep and active	1.56 (1.50–1.63)	1.28 (1.22–1.35)	1.28 (1.23–1.34)
Short sleep and inactive	3.45 (3.30–3.61)	1.33 (1.26–1.41)	1.38 (1.31–1.44)^e^
Long sleep and active	1.87 (1.78–1.96)	1.23 (1.16–1.30)	1.18 (1.12–1.24)
Long sleep and inactive	3.82 (3.63–4.03)	1.24 (1.17–1.32)	1.29 (1.22–1.36)

CI, confidence interval; CVD, cardiovascular disease; IPTW, inverse probability of treatment weighting; OR, odds ratio; Ref, reference group.

Model 1 (Unadjusted): Crude associations accounting only for complex survey weights.

Model 2 (Multivariable Adjusted): Adjusted for age, sex, race/ethnicity, education level, annual household income, and marital status.

Model 3 (IPTW Analysis): A doubly robust estimator model. Weights were derived from a propensity score model predicting physical inactivity conditional on all covariates (age, sex, race, education, income, marital status, BMI, smoking, binge drinking, insurance, general health, depression, diabetes, kidney disease, COPD, asthma, arthritis, and cancer). The outcome model was further adjusted for these covariates to ensure robustness against weight estimation errors.

^a^Independent associations were estimated from separate models where sleep duration and physical activity were mutually adjusted for each other.

^b^Estimates for sleep duration in Model 3 represent associations within the IPTW-weighted pseudo-population balanced for physical activity propensity. The IPTW procedure was applied to physical inactivity only. CI derived from: Short Sleep (Estimate = 0.2289, SE = 0.0170); Long Sleep (Estimate = 0.1538, SE = 0.0184).

^c^Estimate represents the average treatment effect (ATE) of physical inactivity on CVD prevalence in the weighted population. CI derived from: Inactive (Estimate = 0.1053, SE = 0.0116).

^d^Joint associations categorize participants into six mutually exclusive groups based on sleep duration and physical activity status.

^e^This joint exposure category represents the highest-risk group and is the primary focus of the main analysis.

### Assessments of biological interaction

**Figure 1:**
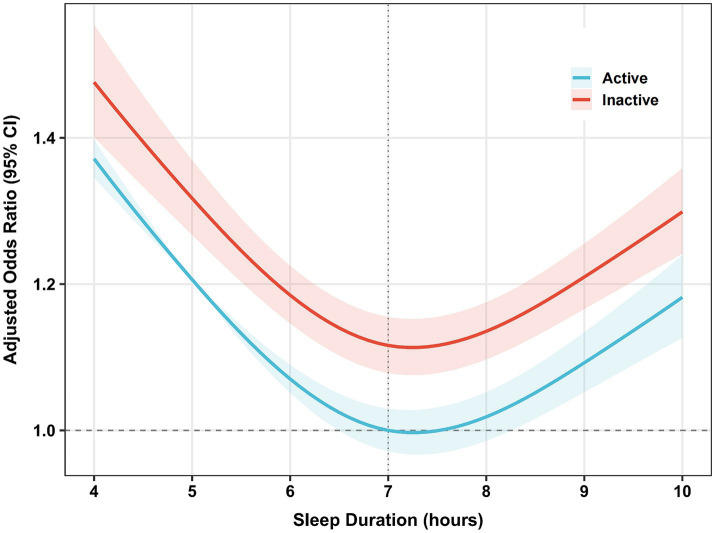
IPTW-adjusted dose–response association of sleep duration with cardiovascular disease stratified by physical activity status.

Although the joint risk was additive, we found no evidence of synergistic interaction on the additive scale. The RERI was −0.03 (95% CI, −0.11 to 0.06), and the Synergy Index was 0.93 (95% CI, 0.75 to 1.15), with confidence intervals crossing the null thresholds (Table 3). This pattern indicates that the adverse effects of short sleep and physical inactivity operate independently and summatively rather than amplifying each other. Consequently, the protective benefit of physical activity remains consistent regardless of sleep duration. This independent relationship was further corroborated by restricted cubic spline analyses (Figure 1), which displayed parallel J-shaped dose–response curves for sleep duration and CVD risk. The curve for inactive participants was shifted vertically upwards compared with active participants across the entire range of sleep duration, illustrating a constant, independent excess risk attributable to inactivity.

**Table 3:** Measures of additive interaction between short sleep duration and physical inactivity on cardiovascular disease risk.

Measures of interaction	Estimate	95% Confidence Interval	*P*-value
RERI	−0.03	−0.11 to 0.06	0.49
AP	−0.02	−0.08 to 0.04	0.52
Synergy index	0.93	0.75 to 1.15	0.5

AP, attributable proportion; CVD, cardiovascular disease; RERI, relative excess risk due to interaction.

Indices were calculated based on the IPTW-weighted doubly robust model (Model 3) adjusting for all covariates listed in Table 1.

### Subgroup analysis and public health impact

**Figure 2:**
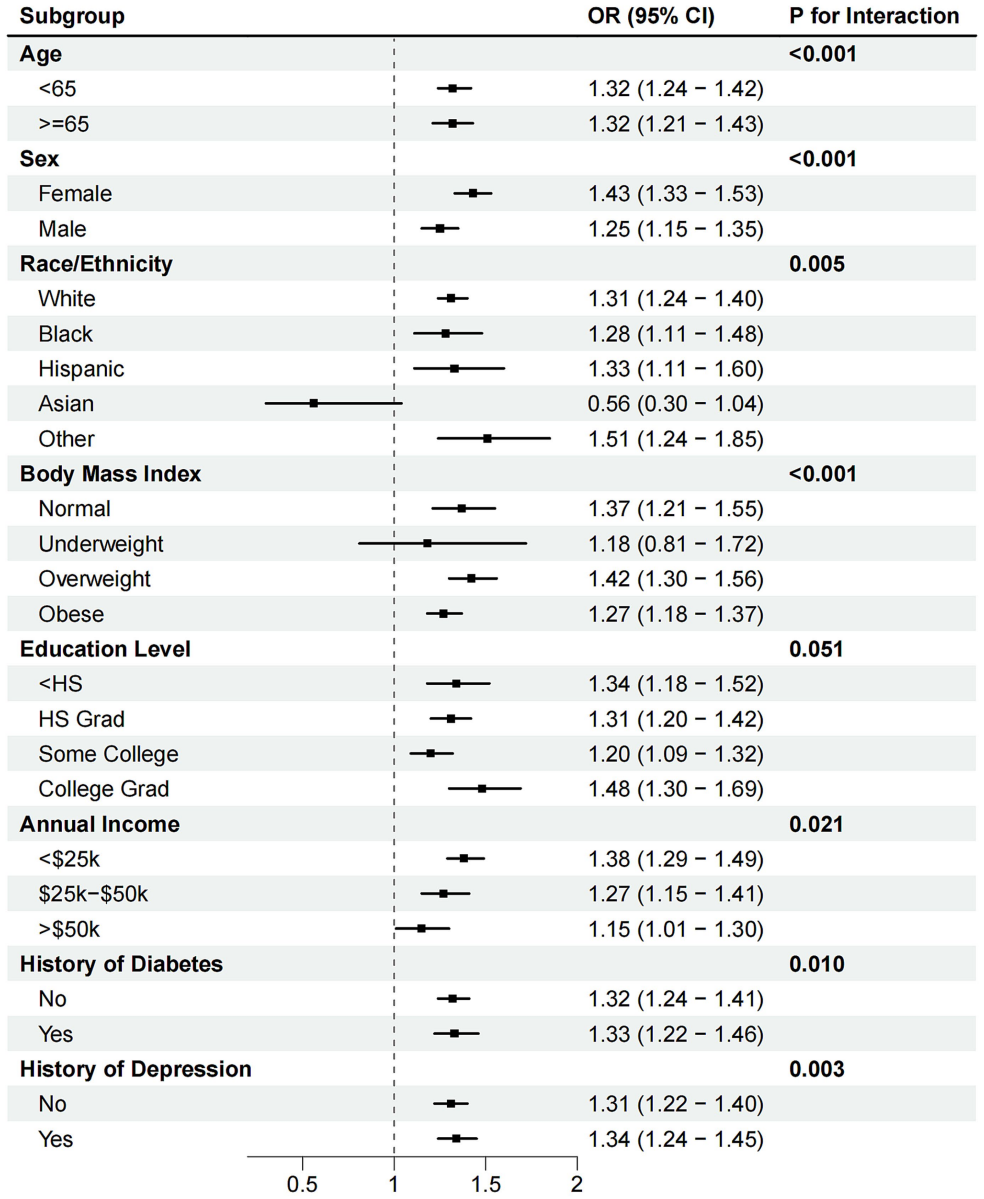
Subgroup analyses of the association between the high-risk joint lifestyle and cardiovascular disease prevalence. Forest plot displays adjusted odds ratios (ORs) and 95% CIs for the joint effect of short sleep and physical inactivity compared with the low-risk reference (normal sleep and active). Estimates are derived from survey-weighted, IPTW-adjusted logistic regression models accounting for all covariates. BMI indicates body mass index; HS, high school; OR, odds ratio; CI, confidence interval.

Stratified analyses revealed that the association between the high-risk lifestyle profile and CVD was universally present across most population strata, though the magnitude varied (Figure 2). Significant multiplicative interactions were observed for sex and BMI (*P_interaction_* < 0.001), with stronger associations noted in females (OR, 1.43) and individuals with normal weight (OR, 1.37). Despite these variations, the risk estimates remained statistically significant in nearly all subgroups. Notably, the Asian subgroup showed a point estimate below unity (OR, 0.56; 95% CI, 0.30–1.04), though the wide confidence interval crossed the null, indicating substantial uncertainty. This imprecision is likely attributable to the relatively small sample size in this subgroup, and we refrain from overinterpreting this estimate.

### Sensitivity and robustness

Sensitivity analyses reinforced the validity of the primary findings (Supplementary Table S2). Restricting the analysis to participants with good-to-excellent self-rated health to mitigate reverse causality yielded consistent results (OR, 1.37; 95% CI, 1.25–1.50). The *E*-value for the primary estimate was 2.10 (lower CI limit, 1.95), suggesting moderate robustness to unmeasured confounding; however, given the self-reported nature of our exposures and the absence of several clinically relevant covariates (e.g., diet, OSA, medication use), we acknowledge that residual confounding cannot be fully excluded. Additionally, excluding the 2020 cycle did not materially alter the primary joint effect estimate (Supplementary Table S3), indicating that pandemic-related behavioral changes did not substantially bias our findings. Multiple imputation yielded estimates slightly attenuated but highly consistent with the complete-case analysis. For the primary joint effect (short sleep and physical inactivity), the MI estimate was OR = 1.29 (95% CI, 1.22–1.36), compared with OR = 1.33 (95% CI, 1.26–1.41) from complete-case Model 2 (Supplementary Table S4), indicating that missing data did not materially bias our findings.

Calibration of Model 2 was satisfactory: observed proportions across deciles of predicted risk closely tracked the predicted probabilities, with all observed values lying within the confidence intervals of the predictions (Supplementary Table S5), indicating that the model provides a well-fitted representation of the data.

## Discussion

In this large-scale analysis representing the US adult population, we provide robust evidence that sleep duration and physical activity function as distinct, non-substitutable pillars of cardiovascular health. Using a doubly robust IPTW framework for physical inactivity, we found that the combination of short sleep and physical inactivity confers the highest cardiovascular risk—a 38% increase in odds compared with a healthy lifestyle. Crucially, our interaction analyses and RCS curves reveal an independent additive effect rather than a synergistic one. This implies that the cardiovascular benefits of physical activity are preserved even among short sleepers, yet activity alone cannot fully eliminate the excess risk caused by sleep deprivation.

Our findings extend the growing body of evidence regarding the joint impact of sleep and physical activity on health outcomes. While previous landmark studies, such as those from the UK Biobank and the MJ Cohort, have primarily focused on mortality endpoints (9, 10, 24, 25), our study provides critical insights into the stage of disease morbidity—a more proximal target for primary prevention. For instance, Chen et al. (10) and Liang et al. (24) reported that high levels of physical activity could virtually eliminate the mortality risk associated with short sleep duration, suggesting a strong compensatory effect at the advanced stage of survival. In contrast, our analysis of cardiovascular disease prevalence reveals a more complex picture. We observed that even among physically active individuals, short sleep duration remained associated with a significantly elevated risk of prevalent cardiovascular disease (OR, 1.28), indicating that physical activity may attenuate but not fully negate the pathogenic effects of sleep deprivation on cardiovascular morbidity. This discrepancy highlights a crucial distinction between survival (mortality) and disease development (morbidity). Emerging evidence suggests that while exercise confers physiological resilience that may delay death (25), maintaining sufficient sleep is indispensable for preventing the initial onset of cardiovascular pathology, possibly by regulating arterial stiffness and endothelial function (7, 12). Our findings align with a recent prospective cohort study of 282,473 U. S. adults, which demonstrated that individuals meeting both physical activity and sleep guidelines had significantly lower mortality risks than those meeting only one, underscoring the independent contribution of each behavior (26). Consolidated with our results, this reinforces the concept that sleep and physical activity are distinct, non-substitutable pillars of cardiovascular health, rather than interchangeable compensatory behaviors. Recent prospective studies, such as those by Huang et al. (9) and da Silva et al. (27), have further demonstrated that the mitigating effect of physical activity on sleep-related mortality is largely driven by moderate-to-vigorous intensity activities, suggesting that our conservative binary classification may have underestimated the true potential of exercise in high-risk populations.

The additive effect of concurrent short sleep and physical inactivity observed in our study is biologically plausible and likely mediated through multiple overlapping physiological pathways. Sleep duration and physical activity act as potent regulators of the neuroendocrine and immune systems. Sleep deprivation is known to induce a state of low-grade systemic inflammation, characterized by elevated levels of C-reactive protein (CRP) and interleukin-6 (IL-6), while simultaneously triggering sympathetic overactivity and endothelial dysfunction—key precursors to atherosclerosis (8, 9, 28). Conversely, regular physical activity exerts a potent anti-inflammatory effect and enhances nitric oxide bioavailability, which can theoretically counterbalance these adverse alterations (29, 30). However, when both risk behaviors co-occur, the protective buffering capacity of the body may be overwhelmed. The “double hit” of sleep-induced sympathetic surge and inactivity-induced metabolic stagnation may lead to accelerated vascular aging and plaque formation (31). Recent proteomic studies have further identified shared molecular signatures underlying these behaviors, involving proteins that regulate circadian rhythms and lipid metabolism, which are dysregulated in the presence of both sleep loss and inactivity (32, 33). Furthermore, these behaviors are intrinsically linked through behavioral clustering; fatigue from sleep deprivation has been shown to acutely diminish the motivation and energy required for next-day physical exertion (34, 35), while inactivity can compromise sleep pressure and quality, creating a vicious cycle that exacerbates cardiovascular risk beyond the sum of independent effects (36, 37).

Our use of restricted cubic splines provides granular evidence for a J-shaped relationship between sleep duration and cardiovascular risk, pinpointing 7 h as the optimal nadir. This finding aligns with recent prospective cohorts and Mendelian randomization studies (31, 38), demonstrating that the risk escalates steeply with sleep curtailment (<6 h) but more gradually with sleep extension (>8 h). The steeper risk gradient for short sleep is consistent with genetic evidence suggesting that habitual short sleep acts as a direct physiological stressor causally linked to hypertension and endothelial dysfunction (31, 39). Conversely, the association with prolonged sleep may partly reflect reverse causality or underlying comorbidities, such as depression or fragmented sleep quality, which are often clustered with sedentary behavior and metabolic dysregulation (40, 41). Although reverse causality is a concern for long sleepers, our sensitivity analysis restricted to individuals with good-to-excellent self-rated health (Supplementary Table S2) yielded a materially unchanged estimate (OR, 1.29; 95% CI, 1.19–1.40). While this does not eliminate residual confounding, it suggests that the long sleep-CVD association is not entirely explained by pre-existing poor health. Nonetheless, we cannot exclude the possibility that undiagnosed comorbidities or poor sleep quality (e.g., sleep fragmentation) contribute to this observed association. Notably, our identification of the “Short Sleep and Inactive” phenotype as the highest-risk group (OR, 1.38) highlights the compounded vulnerability of this population segment. Similar high-risk phenotypes have been identified in the UK Biobank and MJ Cohort, where the co-occurrence of sleep disturbances and physical inactivity was associated with the highest mortality hazard (9, 10). This convergence of evidence across populations underscores that these behaviors are not isolated risk factors but part of an integrated “24-h movement behavior” cycle. Consequently, public health interventions targeting cardiovascular disease must evolve from single-behavior recommendations to comprehensive lifestyle prescriptions that simultaneously address sleep hygiene and physical activity engagement.

The observed heterogeneity across subgroups should be interpreted cautiously. While the high-risk lifestyle profile was consistently associated with elevated CVD risk across most strata, the Asian subgroup exhibited a non-significant inverse estimate (OR, 0.56; 95% CI, 0.30–1.04). The wide confidence interval crossing unity suggests that this finding is imprecise and may reflect limited statistical power due to the smaller sample size in this subgroup. Alternatively, it could be influenced by cultural differences in self-reported lifestyle behaviors, differential survival bias, or unmeasured protective factors specific to this population. Given these uncertainties, we do not interpret this as evidence of a genuinely protective effect.

Our study has several notable strengths, including the use of a large, nationally representative sample and the rigorous application of complex survey weighting, which enhances the generalizability of our findings. Furthermore, the use of restricted cubic splines allowed for a precise characterization of the non-linear sleep-CVD relationship.

However, several limitations warrant consideration. First, the cross-sectional design precludes causal inference. While we identified robust associations, we cannot rule out the possibility of bidirectional causality. To mitigate this, we performed stringent sensitivity analyses excluding participants with fair or poor health, yet the potential for residual reverse causality remains. Second, sleep duration was assessed by self-report, which is subject to recall bias and tends to overestimate actual duration—potentially attenuating our estimates. Additionally, the physical activity assessment in BRFSS was restricted to a binary indicator of any leisure-time activity in the past month, without capturing intensity, frequency, or duration. This crude categorization inevitably introduces non-differential misclassification, which typically biases effect estimates toward the null. Consequently, our observed odds ratios, particularly for the joint effect (OR, 1.38), are likely conservative underestimations of the true cardiovascular risk. Moreover, as highlighted by Huang et al. (9) and Da Silva et al. (27), moderate-to-vigorous physical activity may be required to counteract the harms of short sleep; however, our binary measure precluded such stratification, representing a key data-driven limitation of this study. Third, data availability constraints inherent to the BRFSS survey design influenced our sampling strategy and covariate adjustment. We utilized data exclusively from five even-numbered years (2014–2022) because the sleep duration module was not consistently included in the core questionnaire during odd-numbered years. Consequently, due to the rotating core design of the BRFSS, data on certain specific cardiovascular risk factors, such as hyperlipidemia and hypertension, were not uniformly available in these cycles and thus could not be included in the multivariable adjustment models. Although these factors may lie on the causal pathway between lifestyle behaviors and CVD, they are also established independent risk factors for CVD, and their absence from our models represents additional residual confounding that we cannot fully quantify. Fourth, residual confounding from unmeasured factors—such as dietary patterns, obstructive sleep apnea, and medication use—cannot be completely eliminated. These variables were not captured in the BRFSS core questionnaire and may contribute to residual confounding beyond what we have adjusted for. Fifth, the BRFSS data did not allow for the differentiation of specific sleep disorders (e.g., distinguishing insomnia from obstructive sleep apnea), which limits our ability to identify which aspects of sleep disturbance drive the observed associations. Sixth, by aggregating myocardial infarction, coronary heart disease, and stroke into a single composite outcome, we assumed a shared common pathway, which may have obscured disease-specific etiological heterogeneities. For instance, the association between sleep apnea and stroke may differ in magnitude from its association with coronary artery disease. Our composite endpoint was chosen to enhance statistical power for subgroup inference, but we acknowledge that this approach limits clinical granularity. Seventh, the IPTW procedure was applied only to physical inactivity, while sleep duration was adjusted via regression. Although both exposures were included in the same model, the asymmetry in weighting should be considered when interpreting the joint effect estimates. In addition, although the overall sample was large, subgroup analyses—particularly for racial/ethnic minorities such as Asian participants—were limited by smaller sample sizes, resulting in wide confidence intervals and imprecise estimates. These subgroup findings should therefore be interpreted as exploratory. Finally, although our primary analysis was based on complete cases, a multiple imputation sensitivity analysis yielded estimates highly consistent with the complete-case results (Supplementary Table S4), suggesting that missing data did not substantially bias our findings. Nonetheless, we cannot fully exclude the possibility of residual confounding from unmeasured factors associated with missingness, and our findings may be somewhat less generalizable to populations with lower socioeconomic position.

## Conclusion

Both short and long sleep durations, together with physical inactivity, are independent, additive risk factors for cardiovascular disease. Their combination exerts a cumulative deleterious effect that accounts for a substantial disease burden in the US population. Effective cardiovascular prevention requires a dual-focus approach: promoting physical activity while simultaneously prioritizing sleep health, recognizing that neither behavior can fully substitute for the other.

## Data Availability

The original contributions presented in the study are included in the article/Supplementary material, further inquiries can be directed to the corresponding authors.
