# Supplementary material for: Independent and joint associations of sleep duration and physical activity with cardiovascular disease: a cross-sectional study of 1.6 million US adults
Source: Front Public Health. 2026 Jul 28;14:1886068. doi: 10.3389/fpubh.2026.1886068 (PMC13457713; doi:10.3389/fpubh.2026.1886068)
Supplement: Supplementary file 1 [file Data_Sheet_1.docx]

**Supplementary Materials**

**Tables**

**Supplementary Table S1.** Temporal Trends in the Weighted Prevalence of Cardiovascular Disease, Short Sleep Duration, and Physical Inactivity Among US Adults, BRFSS 2014–2022

**Supplementary Table S2.** Sensitivity Analysis of the Association Between Joint Lifestyle and Cardiovascular Disease After Excluding Participants With Fair or Poor Self-Rated Health

**Supplementary Table S3.** Sensitivity Analysis Excluding the 2020 BRFSS Cycle

**Supplementary Table S4.** Comparison of Complete-Case and Multiple Imputation Estimates for the Joint Associations of Sleep Duration and Physical Activity with CVD (Model 2)

**Supplementary Table S5.** Calibration of the Multivariable-Adjusted Model (Model 2) by Deciles of Predicted Risk

**Figure**

**Supplementary Figure S1.** Flowchart of Cohort Derivation from the BRFSS 2014–2022 Pool

**Supplementary Figure S2.** Love plot for assessing covariate balance before and after Inverse Probability of Treatment Weighting.

**Supplementary Table S1. Temporal Trends in the Weighted Prevalence of Cardiovascular Disease, Short Sleep Duration, and Physical Inactivity Among US Adults, BRFSS 2014–2022**

| **Year** | **CVD Prevalence %(95% CI)** | **Short Sleep (<6 h) %(95% CI)** | **Physical Inactivity %(95% CI)** |
| --- | --- | --- | --- |
| **2014** | 8.7 (8.5-8.9) | 11.7 (11.5-11.9) | 22.4 (22.1-22.7) |
| **2016** | 8.6 (8.4-8.8) | 11.4 (11.1-11.6) | 22.7 (22.4-23.0) |
| **2018** | 8.9 (8.7-9.2) | 11.9 (11.7-12.2) | 22.7 (22.4-23.1) |
| **2020** | 8.5 (8.3-8.8) | 10.7 (10.4-11.0) | 21.9 (21.6-22.3) |
| **2022** | 9.2 (9.0-9.4) | 12.3 (12.0-12.6) | 21.9 (21.5-22.2) |

**Supplementary Table S2. Sensitivity Analysis of the Association Between Joint Lifestyle and Cardiovascular Disease After Excluding Participants With Fair or Poor Self-Rated Health**

| **Joint Lifestyle Category** | **Odds Ratio (95% CI)** | **P Value** |
| --- | --- | --- |
| **Normal Sleep & Active (Reference)** | 1.00 [Reference] | NA |
| Normal Sleep & Inactive | 1.11 (1.07-1.16) | <.001 |
| Short Sleep & Active | 1.32 (1.24-1.40) | <.001 |
| **Short Sleep & Inactive** | **1.37 (1.25-1.50)** | **<.001** |
| Long Sleep & Active | 1.25 (1.16-1.35) | <.001 |
| Long Sleep & Inactive | 1.29 (1.19-1.40) | <.001 |

**Note:**
Data are expressed as weighted odds ratios and 95% confidence intervals (CIs).
The analysis was restricted to participants who reported their general health status as “Excellent,” “Very Good,” or “Good” (n = 1,310,151), excluding those with “Fair” or “Poor” health to mitigate reverse causality bias. The model was fully adjusted for age, sex, race and ethnicity, education level, annual household income, marital status, health insurance, smoking status, alcohol consumption, body mass index, and history of diabetes, hypertension, COPD, kidney disease, depression, arthritis, and cancer.
The highest-risk estimate is indicated in **bold**. Sleep duration categories: Short (<6 h), Normal (6–8 h), Long (>8 h). Physical activity status: Active (any leisure-time physical activity in the past month) vs Inactive. All P values are 2-sided.

**Supplementary Table S3.** Sensitivity Analysis Excluding the 2020 BRFSS Cycle

| Joint Lifestyle Category | OR (95% CI) * | OR (95% CI) ** |
| --- | --- | --- |
| Normal Sleep & Active | 1.00 (Reference) | 1.00 (Reference) |
| Normal Sleep & Inactive | 1.12 (1.09–1.15) | 1.12 (1.09–1.15) |
| Short Sleep & Active | 1.28 (1.23–1.34) | 1.28 (1.23–1.34) |
| **Short Sleep & Inactive** | **1.38 (1.31–1.44)** | **1.37 (1.30–1.43)** |
| Long Sleep & Active | 1.18 (1.12–1.24) | 1.18 (1.12–1.24) |
| Long Sleep & Inactive | 1.29 (1.22–1.36) | 1.28 (1.21–1.35) |

*Main analysis (2014–2022 pooled)

**Sensitivity analysis excluding 2020

Estimates are from the fully adjusted Model 3 (IPTW with doubly robust regression). Excluding the 2020 cycle did not materially change any point estimates, and all confidence intervals remained virtually identical.

**Supplementary Table S4.** Comparison of Complete-Case and Multiple Imputation Estimates for the Joint Associations of Sleep Duration and Physical Activity with CVD (Model 2)

| Exposure Group | Complete-Case Analysis (Model 2) | Multiple Imputation (Model 2) |
| --- | --- | --- |
| Normal Sleep & Active | 1.00 (Reference) | 1.00 (Reference) |
| Normal Sleep & Inactive | 1.10 (1.06 – 1.13) | 1.07 (1.04 – 1.11) |
| Short Sleep & Active | 1.28 (1.22 – 1.35) | 1.25 (1.19 – 1.32) |
| **Short Sleep & Inactive** | **1.33 (1.26 – 1.41)** | **1.29 (1.22 – 1.36)** |
| Long Sleep & Active | 1.23 (1.16 – 1.30) | 1.21 (1.14 – 1.28) |
| Long Sleep & Inactive | 1.24 (1.17 – 1.32) | 1.21 (1.14 – 1.28) |

Data are odds ratios (95% confidence intervals). Estimates derived from multiple imputation by chained equations (MICE) with 5 imputed datasets and 20 iterations, adjusted for all covariates in Model 2 (age, sex, race/ethnicity, education, income, marital status).

| **Decile** | **N (Unweighted)** | **Mean Predicted Probability** | **Observed Proportion** | **95% CI** |
| --- | --- | --- | --- | --- |
| 1 | 157,759 | 0.032 | 0.031 | (0.028 – 0.034) |
| 2 | 157,759 | 0.048 | 0.046 | (0.043 – 0.049) |
| 3 | 157,759 | 0.061 | 0.059 | (0.056 – 0.062) |
| 4 | 157,759 | 0.074 | 0.073 | (0.070 – 0.076) |
| 5 | 157,759 | 0.088 | 0.087 | (0.084 – 0.090) |
| 6 | 157,759 | 0.103 | 0.105 | (0.102 – 0.108) |
| 7 | 157,759 | 0.120 | 0.122 | (0.118 – 0.126) |
| 8 | 157,759 | 0.140 | 0.143 | (0.139 – 0.147) |
| 9 | 157,759 | 0.168 | 0.171 | (0.166 – 0.176) |
| 10 | 157,759 | 0.228 | 0.234 | (0.228 – 0.240) |

**Supplementary Table S5.** Calibration of the Multivariable-Adjusted Model (Model 2) by Deciles of Predicted Risk

Data are presented as weighted proportions accounting for the complex survey design. The calibration curve based on these data showed good agreement with the 45-degree diagonal line, indicating satisfactory model calibration.

**Supplementary Figure S1.** Flowchart of Cohort Derivation from the BRFSS 2014–2022 Pool

**Supplementary Figure S1.** Love plot for assessing covariate balance before and after Inverse Probability of Treatment Weighting.

The absolute standardized mean differences (SMDs) are shown for key covariates. Red dots represent unadjusted SMDs; blue dots represent SMDs after IPTW adjustment. After weighting, most covariates achieve improved balance, with adjusted SMDs approaching zero, indicating successful reduction in systematic differences between treatment groups. Covariates with SMDs below 0.1 after adjustment are generally considered well-balanced.
